# Isolation of T cell receptor specifically reactive with autologous tumour cells from tumour-infiltrating lymphocytes and construction of T cell receptor engineered T cells for esophageal squamous cell carcinoma

**DOI:** 10.1186/s40425-019-0709-7

**Published:** 2019-08-28

**Authors:** Qin Tan, Chaoting Zhang, Wenjun Yang, Ying Liu, Palashati Heyilimu, Dongdong Feng, Liying Xing, Yang Ke, Zheming Lu

**Affiliations:** 1 Key Laboratory of Carcinogenesis and Translational Research (Ministry of Education/Beijing), Laboratory of Genetics, Peking University Cancer Hospital & Institute, No. 52 Fucheng Road, Beijing, 100142 China; 2 Key Laboratory of Carcinogenesis and Translational Research (Ministry of Education/Beijing), Department of Biochemistry and Molecular Biology, Peking University Cancer Hospital & Institute, No. 52 Fucheng Road, Beijing, 100142 China; 3 Key Laboratory of Fertility Preservation and Maintenance (Ministry of Education), Cancer Institute of the General Hospital, Ningxia Medical University, Yinchuan, Ningxia 750004 People’s Republic of China; 4 Key Laboratory of Carcinogenesis and Translational Research (Ministry of Education/Beijing), Department of Head and Neck Surgery, Peking University Cancer Hospital & Institute, No. 52 Fucheng Road, Beijing, 100142 China

**Keywords:** Tumor-infiltrating lymphocytes (TIL), T cell receptor-engineered T cells (TCR-Ts), Esophageal squamous cell cancer (ESCC), Autologous tumor cells, CD137

## Abstract

**Background:**

T cell receptor-engineered T cells (TCR-Ts) therapy is a promising cancer treatment strategy. Nowadays, most studies focused on identification of high-avidity T cell receptors (TCRs) directed against neoantigens derived from somatic mutations. However, few neoantigens per patient could induce immune response in epithelial cancer and additionally many tumor-specific antigens could be derived from noncoding region. Autologous tumor cells (ATCs) could be unbiased stimulators in activating and enriching tumor-reactive T cells. However, it’s unknown if T cells engineered to express TCRs isolated from tumor-reactive T cells enriched by ATCs have strong antitumor response.

**Methods:**

In this study, multiple TIL fragments obtained from a patient with esophageal squamous cell carcinoma (ESCC) were screened for specific recognition of ATCs. Tumor-reactive TILs were enriched by in vitro repeated stimulation of ATCs and isolated based on CD137 upregulation. Subsequently, tumor-reactive TCR was obtained by single-cell RT-PCR analysis and was introduced into peripheral blood lymphocytes to generate TCR-Ts.

**Results:**

We found that phenotype and effect function of TIL fragments derived from different tumor sites were spatially heterogeneous. Of four TIL fragments, only TIL-F1 could specifically identify ATCs. Subsequently, we isolated CD8^+^ CD137^+^ T cells from pre- and post-stimulated TIL-F1 co-cultured with ATCs, and identified their most dominant TCR. This TCR was introduced into PBLs to generate TCR-Ts, which specifically identified and killed ATCs in vivo and in vitro.

**Conclusion:**

This strategy provides the means to generate tumor-reactive TCR-Ts for ESCC, which is especially important for patients without prior knowledge of specific epitopes and might be applied for other cancers.

**Electronic supplementary material:**

The online version of this article (10.1186/s40425-019-0709-7) contains supplementary material, which is available to authorized users.

## Background

Esophageal cancer is one of the most common cancers worldwide, with higher incidence rates in Eastern Asia and in Eastern and Southern Africa, in which esophageal squamous cell carcinoma (ESCC) is the predominant histologic type [1]. Despite advances in diagnosis and treatment, the prognosis of advanced ESCC remains poor, due to its invasive and diffuse nature [2]. Thus, new effective treatment strategies are urgently needed.

Adoptive cell therapy including T cell receptor-engineered T cells (TCR-Ts) has mediated effective anti-tumor responses in several cancers [3]. Tumor-specific TCR, which is critical for anti-tumor efficacy of TCR-Ts, could be isolated from T cells stimulated and activated by tumor-specific antigens. Some studies reported that neoantigens derived from somatic mutations could activate and enrich tumor-reactive T cells, which could mediate objective clinical responses [4–6]. However, in general, few neoantigens per patient could induce immune response in epithelial cancer [7] and additionally recent study reported that many tumor-specific antigens were derived from noncoding region [8], which suggested urgent need to find more tumor-reactive antigens, especially for cancers with low mutation burden. Autologous tumor cells (ATCs) expressing various tumor antigens could be unbiased stimulators in activating and enriching tumor-reactive T cells [9–11].

CD137 is a T cell activation marker and thus upregulation of CD137 expression on activated T cells could be used to identify and isolate tumor-reactive T cells [12, 13]. In this study, we attempted to use CD137 upregulation on tumor infiltrating lymphocytes (TILs) in vitro stimulated with autologous tumor cells to identify tumor-reactive T cells and subsequently isolate their TCRs which were then introduced into PBLs to generate tumor-reactive TCR-Ts. The specific recognition and killing capacity of TCR-Ts against autologous tumor cells in vivo and in vitro were observed and evaluated.

This strategy provides the means to generate tumor-reactive TCR-Ts for a truly tumor-specific ESCC treatment, which might also be applied for treatment of other cancers.

## Methods

### Patient samples

Tumor sample from a 63-year-old woman with metastasis ESCC from Peking University Cancer Hospital was obtained in our preclinical research with informed consent. The tumor sample was collected in tubes containing sterile saline solution and resected into several fragments for a) TIL culture; b) generation of patient-derived xenograft (PDX) model. This study was approved by the Institutional Review Board of the Peking University School of Oncology, China.

### Cell lines and monoclonal antibodies

HEK 293-FT (Life Technologies), a packaging cell line used to produce high-titer lentivirus supernatants, were cultured in complete Dulbecco’s Modified Eagle Medium (DMEM, Gibco, USA) supplemented with 10% fetal bovine serum(FBS, Gibco, USA) containing 0.1 mM MEM Non-Essential Amino Acids, 1 mM sodium pyruvate and 2 mM Glutamax (Life Technologies, USA) at 37 °C with 5% CO2.

Flow cytometry staining antibodies were shown as below: CD3-BUV395(Clone: UCHT1), CD4-PE-CF594(Clone: RPA-T4), CD8-PE-Cy7(Clone: RPA-T8), PD-1-BUV737(Clone: EH12.1), CCR7-PE(Clone: 150503), CD45RA-APC(Clone: HI100), CD137-APC(Clone: 4B4–1), CD107A-AF647(Clone: H4A3), CD107b-AF647(Clone: H4B3), Fixable Viability Stain 780 (FVS780). All antibodies were from BD Biosciences, except for anti-mouse TCR-β constant region (clone H5–597, eBioscience, USA). The blocking antibody against HLA class I were from eBioscience (clone: W6/32).

### HLA typing for the patient and donors

DNA of autologous tumor cells or the patient or donors’ peripheral blood were exstracted with DNeasy Blood & Tissue Kit (QIAGEN, Germany) according to the manufacturer’s protocol. Genotyping of HLA alleles was performed using high-resolution, high-throughput HLA genotyping with deep sequencing (BGI Diagnosis, Shenzhen, China). The HLA types of the patient and donors were outlined in Additional file 2: Table S1.

### Generation of TILs, PDX models and autologous tumor cells

TILs were generated as previous described [14, 15] with slightly modification. Briefly, tumor sample was minced into approximately 1–2 mm fragments and each fragment was placed into a well of a 24-well plate comprised of T cell media and 50 ng/mL OKT3 antibody (ACRO, USA). T cell media consisted of X-VIVO 15 (Lonza, USA); Glutamax (Life Technologies, USA); IL-2 (50 U/mL, Perprotech, USA), IL-7 (10 ng/mL, Perprotech, USA); IL-15(10 ng/mL, Perprotech, USA). T cells were incubated at 37 °C with 5% CO_2,_ and passaged to maintain a density of 1 × 10^6^ cells/mL until there were enough TILs used for screening of tumor-specific T cells.

PDX model was established by implanting tumor fragments mixed with matrigel subcutaneously into immunodeficient NOD-SCID mice to generate PDX model. ESCC PDX models could be promising for individual therapy, since we have verified that the molecular characteristics of ESCC PDXs were in accordance with primary patient tumors in our previous study [16].

Autologous tumor cells were generated from tumor specimens based on successfully established PDX model [17]. Dissected tumor samples were dissociated into single-cell suspension, using the human Tumor Dissociation Kit (Miltenyi Biotech, Germany) with gentleMACS Octo Dissociator with Heaters (Miltenyi Biotech, Germany) according to the manufacturer’s instructions. Single-cell suspensions were harvested, washed with 1 × phosphate-buffered saline (PBS), and then resuspended in 6-well plate with complete media (DMEM, 20% FBS, 2 mM Glutamax) at 37 °C with 5% CO_2_.

### TILs phenotypic characterization by flow cytometry

Single-cell pellets of TILs were stained with CD3-BUV395, CD4-PE-CF594, CD8-PE-Cy7, CCR7-PE, CD45RA-APC, PD-1-BUV737 antibody cocktails. Cells were washed with PBS prior to acquisition on BD FACS Aria III flow cytometer. Data files were analyzed using FlowJo vX software (FlowJo, Tree Star), gated on single cells. Dead cells and debris were excluded by staining with Fixable Viability Stain 780 (FVS780). Phenotypes of T cells were gated on total CD3^+^ T cells.

### Initial screening of TILs for recognition of autologous tumor cells

Autologous tumor cells were pretreated with complete media containing DAC cocktails (DAC (10 μM, Sigma Aldrich, USA), IFNγ (100 U/mL, ACRO, USA) and TNF-α(10 ng/mL, ACRO,USA) for 48 h, which restored cell surface expression of HLA molecules through enhancing the mRNA expression of HLA-related molecular including TAP or LMP genes or inhibiting DNA methylation [18–20]. Both IFN-γ enzyme-linked immunospot (ELISPOT) and enzyme-linked immunosorbent assays (ELISA) were used to screen tumor-reactive TILs cocultured with pretreated ATCs.

### IFN-γ ELISPOT assay

Human IFN-γ ELISPOT Kit (with precoated plates, Abcam, USA) was performed as the protocol’s procedure. Briefly, 2 × 10^4^ T cells, rested in cytokines-free media overnight, and 1 × 10^4^ PBS-washed autologous tumor cells were incubated together for approximately 20 h in the absence of exogenous cytokines at 37 °C with 5% CO_2_. The number of colored spots was determined by ImmunoSpot plate reader and associated software (Celluar Technologies, USA).

### IFN-γ ELISA assay

1 × 10^6^ responder cells (T cells) and 1 × 10^5^ target cells (ATCs) were incubated together in a 96-well plate in the absence of exogenous cytokines for 18-24 h. Coculture supernatant was transferred into a new 96-well plate, and IFN-γ concentration was measured by using commercially available human IFN-γ ELISA kit (ExCell Bio, China) as manufacturer’s protocols. In HLA blocking experiment, ATCs were pretreated with HLA-blocking antibody (clone W6/32, 50 μg/mL). Following a 3.5-h incubation, 5 × 10^4^ target cells (ATCs) and 1 × 10^5^ responder cells (T cells) were cocultured overnight for assessment of IFN-γ releasing level with the standard IFN-γ ELISA procedure [21].

### Cytotoxic assay

CFSE-based cytotoxity assay was performed as previously described [22, 23] with slightly alteration. Target cells were labeled with 5 μM CFSE (BD Biosciences) for 10 min and then cocultured with TCR-Ts at 37 °C for 4 h, at E: T ratio of 1:1 and 4:1. After the coculture, 1 μg/mL propidium iodide (PI, BD Biosciences) was added for assigning the ratio of cell death, and the samples were analyzed by flow cytometry.

### Enrichment and isolation of tumor-reactive TILs after repeated stimulation with autologous tumor cells

For enrichment of tumor-reactive TILs, 2 × 10^6^ TIL-F1 and TIL-F4 were stimulated with 2 × 10^5^ autologous tumor cells pretreated with DAC cocktails in T-cell media for 1 week, respectively, after which they were stimulated with autologous tumor cells one more time. Moreover, both pre- and post-stimulated TILs were cocultured with 1 × 10^5^ autologous tumor cells (E: T = 5:1) to evaluate their ability of specifically identifying and killing autologous tumor cells using flow cytometry. Data were analyzed using FlowJo vX software (Treestar Inc) after gating on live cells (FVS780 negative). Meanwhile, CD8^+^CD137^+^ T cells of pre- and post-stimulated TIL-F1 were sorted into 96-well PCR plates by single-cell sorting (Additional file 1: Figure S3). And then 96-well PCR plates were immediately put into liquid nitrogen and conserved in minus 80°C prior to running single-cell RT-PCR.

### T cell receptor sequencing and analysis

For single-cell PCR, all the primer sequences were listed in Additional file 2: Table S2 as previously described [24] except that the primers of TRBC were optimized, the final concentration of each Vα and Vβ region primers was 5 μM, and final concentration of TRAC and TRBC primers was 20 μM. The single-cell RT-PCR reaction condition for first-step RT-PCR was as follows: 30 min at 50 °C for RT reaction; 95°Cfor 15 min and 30 cycles of 94 °C for 30s, 52 °C for 30s, 72°C for 1 min; 72°C for 10 min. For the second cycle, 2 μL of cDNA product was used as template for TCRα/β separately in total 20 μL 2nd-PCR TRA/TRB mix (Additional file 2: Table S3), containing multiple internal primer sequences (INT) of Vα/Vβ and one primer for Cα/Cβ, with PrimeSTAR® HS DNA Polymerase (Takara Bio, Japan). The cycling program was 98 °C for 1 min; 98 °C for 10s,52°C for 10s,72°C for 45 s × 43;72 °C for 10 min.The second PCR products were analyzed with TRA sequencing primer for TCRα or TRB sequencing primer for TCRβ (Additional file 2: Table S3). PCR products were purified and sequenced by Sanger sequencing method. The TCR sequences were analyzed using IMGT/V-Quest tool (http://www.imgt.org/).

### Construction of lentivirus vectors and transduction of PBLs

TCRα/β chains were synthesized (GenScript) and cloned into the lentivirus vector. TCR was constructed in a β/α chain order and its constant regions were replaced by mouse counterparts modified with interchain disulfide bond and hydrophobic substitution as previously described, which not only was convenient for detection of TCR-T, but also improved TCR pairing and TCR/CD3 stability [17, 25]. However, since murine constant region of TCR-T could potentially be immunogenic in clinical application, human constant region could be essential for construction of TCR in order to improve the longevity of TCR-T persistence and enhance their therapeutic efficacy in patients.

Transduction of PBLs was conducted as previously delineated [17, 21, 26] with slightly modification. In brief, human peripheral blood mononuclear cells (PBMCs) were separated by centrifugation on a Ficoll-Paque Plus (GE Healthcare, USA) and then stimulated in T-cell media with 50 ng/ml OKT3 and 1 μg/ml anti-CD28 for 2 days before transduction. TCR lentivirus were generated by cotransfection of 293-FT cells with lentivector and packaging plasmids (ratio of pLP1: pLP2:pVSV-G is 2:2:1) using PEI MAX 40000 (Polysciences Inc. USA) [27]. The lentiviral supernatants were harvested at 48 and 72 h after transfection and concentrated using optimized ultracentrifugation approaches with 20,000 g, 90 min at 4 °C [28]. Activated T cells were transduced by concentrated lentivirus at the presence of 8 μg/mL polybrene (Sigma-Aldrich, USA). The transduction efficiency was assessed by flow cytometry using mouse TCR-β chain constant region staining.

### Treatment of PDX models by TCR-Ts

NOD/SCID mice were used to establish patient-derived xenograft approved by the Institutional Review Board of the Peking University School of Oncology, China. Tumor treatment was on days 5 and 12 following tumor inoculation and consisted of two intravenous injections of 4 × 10^6^ T cells as well as a single intraperitoneal injection of 5 mg/kg DAC on day 5. Tumor size was determined by caliper measurement of perpendicular diameters of each tumor and was calculated using the following formula: tumor volume (mm^3^) = [(length) × (width) × (width)]/2.

### Statistics analysis

Statistical analysis was performed using GraphPad Prism 7.0 (GraphPad Software, CA) and SPSS software (version 24; IBM SPSS, Armonk, NY, USA). Statistical comparison was conducted with Student’s t test, and two-way repeated measures ANOVA. All tests were two-sided and *p* value < 0.05 was considered statistically significant. All in vitro experiments were performed more than three independent experiments.

## Results

### Phenotype and functional screening of different TIL fragments

Tumor specimen was obtained from a 63-year-old woman with ESCC. Clinical characteristics and HLA types of the patient are outlined in Additional file 2: Table S1. In order to screen tumor-reactive TILs, we obtained four TIL fragments (TIL-F1 to TIL-F4) from different areas in one resected lesion.

**Fig. 1:**
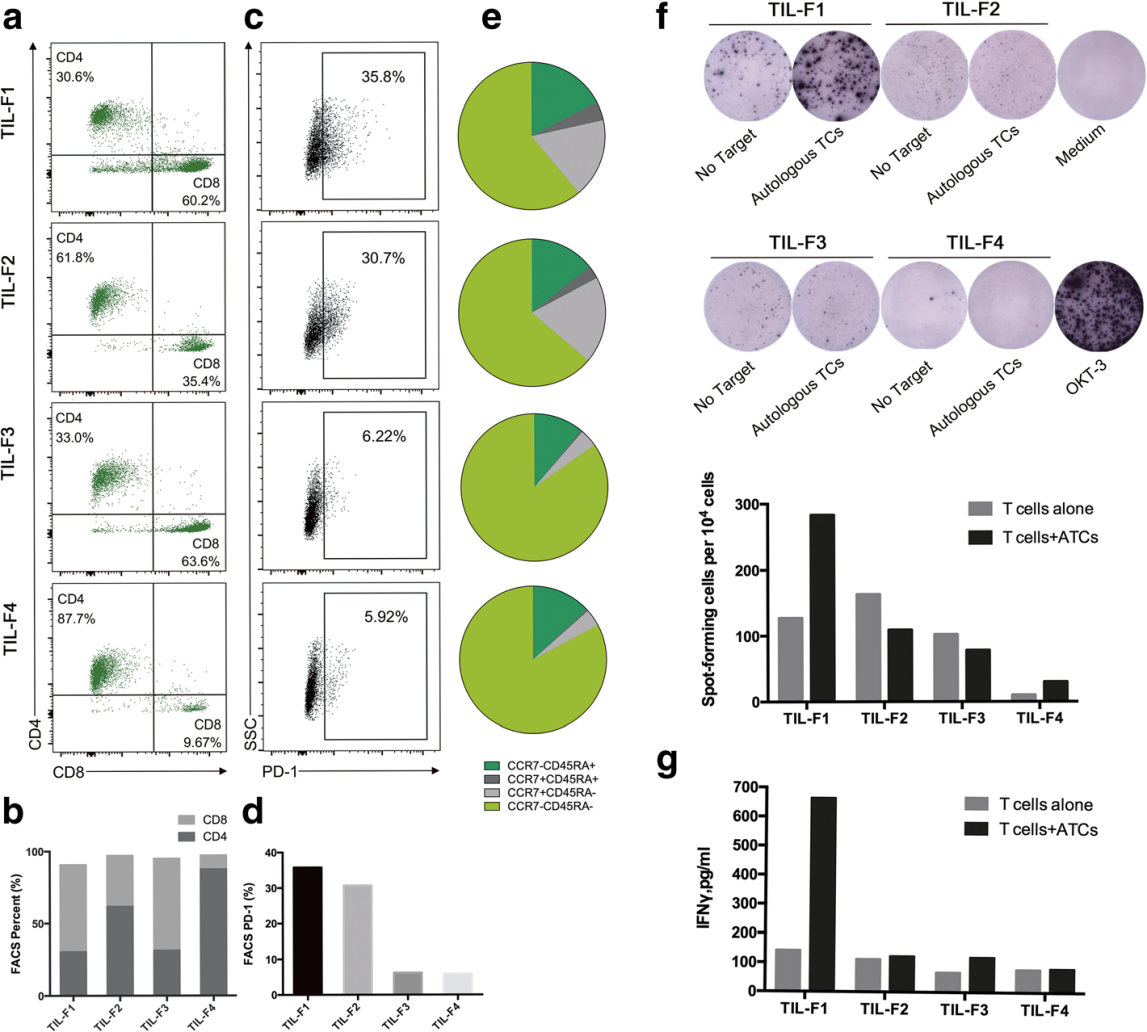
Phenotype and functional screening of different tumor infiltrating lymphocytes (TILs) fragments. **a** Flow cytometry analysis revealed percentages of CD4^+^ and CD8^+^ T cells from TIL-F1 to TIL-F4. **b** CD4/CD8 ratio. **c** The percentages of PD-1^+^T cells in four TIL fragments. **d** Comparision of PD-1 expression. **e** Comparison of memory-phenotype T cells. **f** IFN-γ ELISPOT analysis of all four TIL fragments cocultured with autologous tumor cells (ATCs). TILs with no targets are negative controls. Medium well is the blank negative control and OKT-3 well is the positive control. Column histogram summarized the number of positive spots. **g** IFN-γ ELISA measurement of all four TIL fragments cocultured with ATCs. T cells with no targets are negative controls. The results shown are representative of independent experiment done with repeated 3 times

To evaluate spatial heterogeneity of TILs, we measured the phenotypic characteristics of four TIL fragments derived from different anatomical sites of tumor sample by flow cytometry. The percentages of CD3^+^ T cells in all four TIL fragments were similar and approximately 99% (Additional file 1: Figure S1). However, percentages of CD4^+^ TILs hugely varied from 30.6 to 87.7%, and percentages of CD8^+^ TILs from 9.67 to 63.6%, suggesting significant difference in distribution of CD4^+^ and CD8^+^ TILs among different anatomical sites (Fig. 1a and b). The level of PD-1 expression hugely varied in four TIL fragments, with higher proportions in TIL-F1 and TIL-F2 (35.8 and 30.7%, respectively; Fig. 1c and d). The percent of effector-memory T cells (CCR7^−^CD45RA^−^) was highest in all four TIL fragments, followed by effector T cells (CCR7^−^CD45RA^+^), as showed in Fig. 1e and Additional file 1: Figure S2.

To screen tumor-reactive TILs, TILs (TIL-F1 to TIL-F4) were separately co-cultured with ATCs and we found TIL-F1 co-cultured with ATCs produced a significantly higher level of IFN-γ than TIL-F1 alone but this finding was not found in TIL-F2 to TIL-F4 by enzyme-linked immunospot (ELISPOT) assay and enzyme-linked immunosorbent (ELISA) assay (Fig. 1f and g). These data suggested that TIL fragments derived from different tumor sites were spatially heterogeneous and additionally TIL-F1 had potential anti-tumor activities.

### Isolation of tumor-reactive TCRs from TIL-F1 based on CD137 expression by in vitro stimulation of ATCs and sorting

**Fig. 2:**
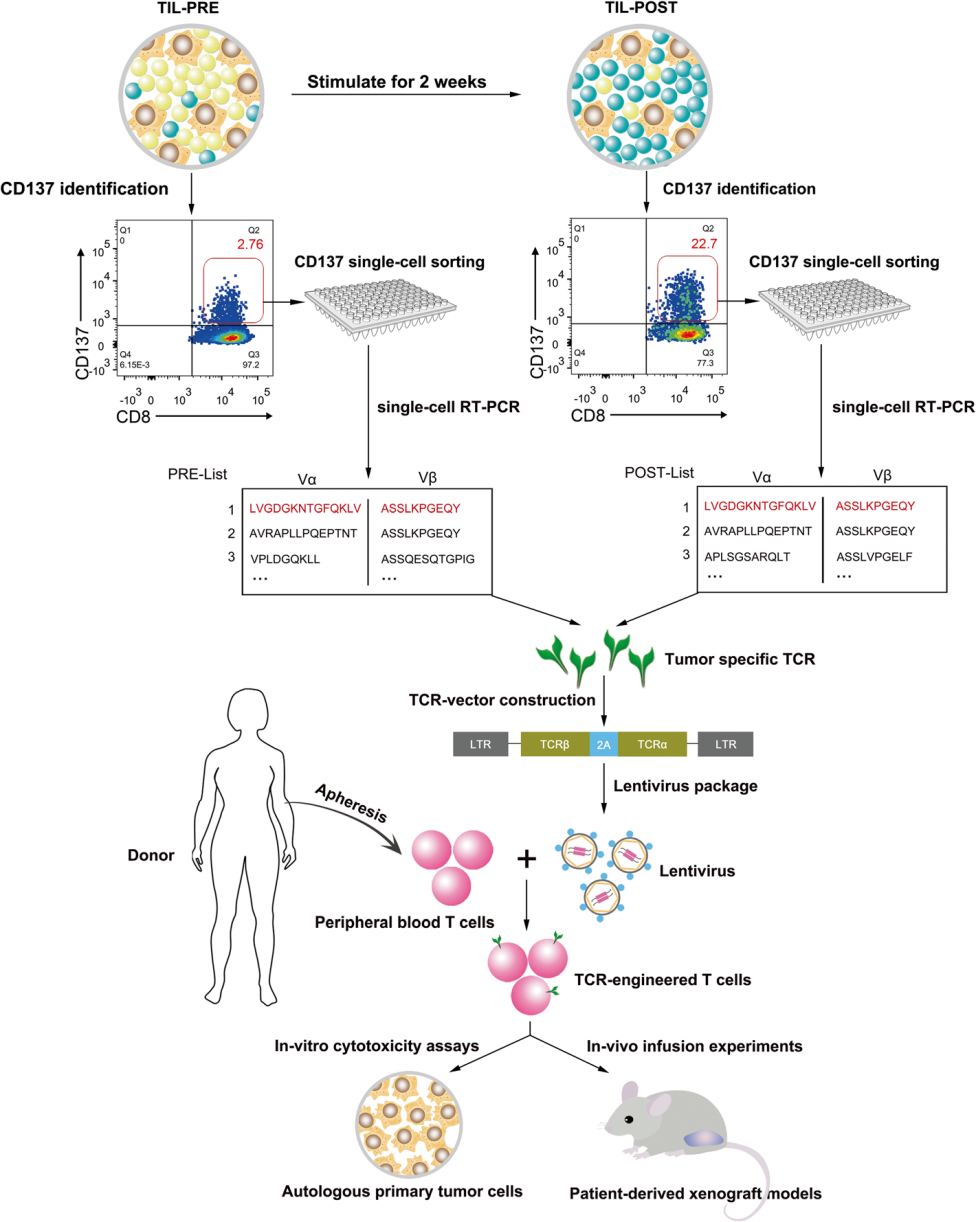
Flowchart for isolation of TCRs from CD137 positive TIL-F1 and function verification of corresponding TCR-Ts. TIL-F1 was stimulated with ATCs for twice, and single CD8^+^ CD137^+^ TIL-F1 before and after stimulation (namely PRE and POST) were both sorted into 96-well plates and were amplified using single-cell RT-PCR to obtain their TCRs. Subsequently, the most dominant TCR in CD8^+^ CD137^+^ TIL-F1 before and after stimulation was cloned into lentiviral vector and introduced into donor peripheral blood lymphocytes (PBLs) to generate tumor-reactive T cell receptors-engineered T cells (TCR-Ts). Finally, we evaluated whether this TCR-Ts could specifically identify and recognize ATCs in vivo and in vitro

**Fig. 3:**
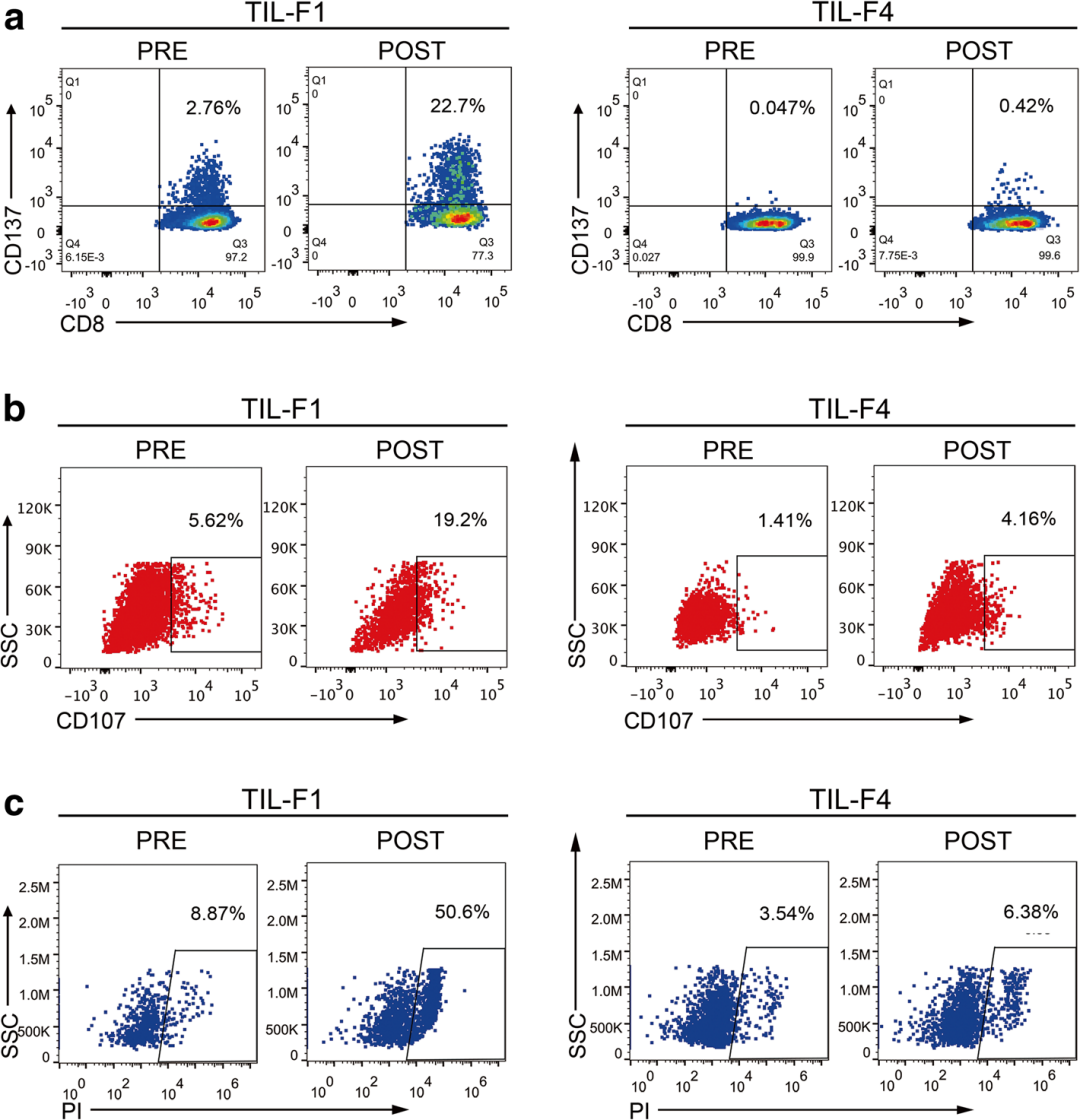
Enrichment of TIL-F1 and TIL-F4 by in vitro repetitive stimulation of ATCs. TIL-F1 and TIL-F4 were stimulated by autologous tumor cells (ATCs) for 1 week, respectively, after which they were stimulated with ATCs one more time. Flow cytometry analysis was used to evaluate specific recognition and cytotoxicity of both pre- and post-stimulated TILs against ATCs by staining cells with CD137 antibody (**a**), CD107 antibody (**b**) and PI antibody (**c**). Data are representative of more than three independent experiments

To further validate anti-tumor activity of TIL-F1 and isolate tumor-reactive TCRs, TIL-F1 was stimulated with ATCs for twice, and single CD8^+^ CD137^+^ TIL-F1 before and after stimulation (namely PRE and POST) were both sorted into 96-well plates and were amplified using single-cell PCR to obtain their TCRs, presented in Fig. 2. Therefore, the most dominant TCR of post-stimulated CD8^+^ CD137^+^ TIL-F1 could be most likely tumor-reactive TCR, which was transduced into donor PBLs to generate tumor-reactive TCR-Ts. To exclude possibility of non-specific amplification of TIL-F1 stimulated with ATCs, TIL-F4 as a negative control was also stimulated with ATCs based on the same stimulation procedure as TIL-F1. The flow cytometry-based assays indicated that the percentage of tumor-reactive T cells in post-stimulated TIL-F1 was significantly higher than that in pre-stimulated TIL-F1 and yet percentages of tumor-reactive T cells in pre- and post-stimulated TIL-F4 were low and similar (Fig. 3).

### Construction and functional validation of tumor-reactive TCR-Ts

**Fig. 4:**
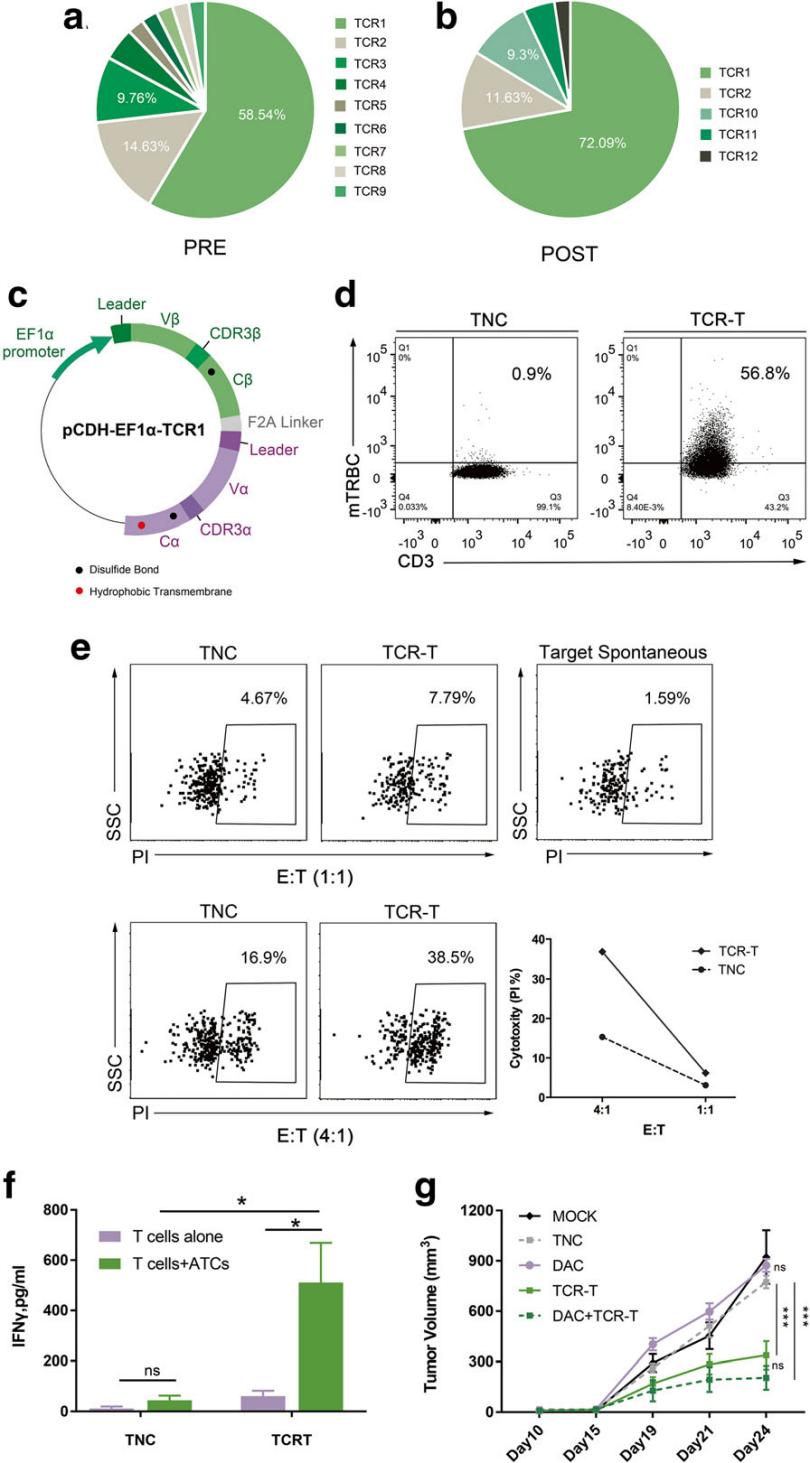
Identification of tumor-specific TCRs and functional verification of corresponding TCR-Ts. TCRs distribution of CD8^+^ CD137^+^ T cells in pre- (**a**) and post-stimulated (**b**) TIL-F1 by single-cell RT-PCR analysis. TCRs sequences are listed with different colors in order from most to least frequent, named TCR1 to TCR12, respectively. **c** Sketch map of pCDH-EF1α-TCR1 lentiviral vector. The construct employed the β-α chain order, added murine constant region, disulfide bond (presented as black dots), and α chain hydrophobic-substitutions (presented as red dot). Leader, leader sequences of TCRα and TCRβ chains, respectively; EF1α promoter, elongation factor 1 alpha promoter; F2A linker, Furin-P2A linker. **d** Transduction efficiencies were measured by staining cells with an anti-murine TCR-β chain constant region antibody. The results were representative of independent experiments done with more than three different donors. **e** Cytotoxicity capacity of TCR-Ts against ATCs. Line chart summarized the cytotoxicity by subtracting the ATCs spontaneous death at different E: T ratios. Data were representative of at least three independent experiments with more than three different donors. **f** IFN-γ ELISA measurement of TCR-Ts and TNC targeting ATCs. The results are representative of more than three independent experiments in more than three different donors (*p < 0.05, Student paried *t* test). **g** Antitumor activity of TCR-Ts against patient derived xenograft models. The tumor volume is plotted on the y axis. Time after tumor cell injection is plotted on the x axis. The mean values from each group are plotted. Error bars represent the SEM (*n* = 5 mice per group, ***p < 0.001, analyzed by two-way repeated measures ANOVA). The results are representative of 2 independent experiments. MOCK, none; TNC, two intravenous injections of untransduced T cells; DAC, a single intraperitoneal injection of DAC; TCR-T, two intravenous injections of TCR-Ts; DAC + TCR-T, two intravenous injections of TCR-Ts and a single intraperitoneal injection of DAC

We isolated approximately 100 CD8^+^ CD137^+^ T cells from pre- and post-stimulated TIL-F1 co-cultured with ATCs. 43 and 42 TCR α/β chain pairs were identified by single-cell TCR sequencing from pre- and post-stimulated CD8^+^ CD137^+^ T cells, respectively (Table 1 and Table 2). The percentages of the first, second and third ranked TCRs in pre-stimulated CD8^+^ CD137^+^ TIL-F1 were 58.54, 14.63, 9.76%, respectively (Table 1, Fig. 4a). After stimulation, the percentage of only the first ranked TCR (namely TCR1) in pre-stimulated TIL-F1 significantly increased (Table 2, Fig. 4b), which suggested that the first ranked TCR could be most likely tumor-reactive TCR.

**Table 1:** TCR genes of CD8^+^CD137^+^T cells in pre-stimulated TIL-F1

ID	TRAV	TRAJ	CDR3 aa seq.	TRBV	TRBD	TRBJ	CDR3 aa seq.	CD8^+^CD137^+^	
								Rank	Freq. (%)
1	TRAV4*01	TRAJ8*01	LVGDGKNTGFQKLV	TRBV7–9*03	TRBD2*01	TRBJ2–7*01	ASSLKPGEQY	1	58.54
2	TRAV1–2*01	TRAJ40*01	AVRAPLLPQEPTNT	TRBV7–9*03	TRBD2*01	TRBJ2–7*01	ASSLKPGEQY	2	14.63
3	TRAV1–2*01	TRAJ16*01	VPLDGQKLL	TRBV3–1*01	TRBD1*01	TRBJ2–5*01	ASSQESQTGPIGGTQY	3	9.76
4	TRAV13–1*02	TRAJ52*01	AASTDNAGGTSYGKLT	TRBV6–6*01	TRBD2*01	TRBJ1–2*01	ASSYGNSGAGGYGYT	4	4.88
5	TRAV8–6*02	TRAJ32*02	AVPVVLQTSS	TRBV7–3*01	TRBD2*01	TRBJ2–1*01	ASSLGGNEQF	5	2.44
6	TRAV1–2*01	TRAJ15*01	AVRETGQAGTALI	TRBV28*01	TRBD1*01	TRBJ2–7*01	ASRLDRASSYEQY	6	2.44
7	TRAV21*01	TRAJ6*01	AAGIRRKLHTY	TRBV19*01	TRBD1*01	TRBJ1–1*01	ASSITGRTEAF	7	2.44
8	TRAV41*01	TRAJ45*01	AVSRGHDSGGGADGLT	TRBV6–5*01	TRBD1*01	TRBJ2–7*01	ASSYGDSYEQY	8	2.44
9	TRAV19*01	TRAJ53*01	ALSLNSGGSNYKLT	TRBV7–9*03	TRBD2*01	TRBJ2–1*01	ASSPVLNEQF	9	2.44

*TCR* T-cell receptor, *CDR3* Complementarity-determining region, *V* Variable, *D* Diversity, *J* Joining, *TRAV* T-cell receptor Vα, *TRBV* T-cell receptor Vβ

*subtypes of TCR genes

**Table 2:** TCR genes of CD8^+^CD137^+^T cells in post-stimulated TIL-F1

ID	TRAV	TRAJ	CDR3 aa seq.	TRBV	TRBD	TRBJ	CDR3 aa seq.	CD8^+^CD137^+^	
								Rank	Freq. (%)
1	TRAV4*01	TRAJ8*01	LVGDGKNTGFQKLV	TRBV7–9*03	TRBD2*01	TRBJ2–7*01	ASSLKPGEQY	1	72.09
2	TRAV1–2*01	TRAJ40*01	AVRAPLLPQEPTNT	TRBV7–9*03	TRBD2*01	TRBJ2–7*01	ASSLKPGEQY	2	11.63
10	TRAV13–1*02	TRAJ22*01	APLSGSARQLT	TRBV7–9*03	No Result	TRBJ2–2*01	ASSLVPGELF	3	9.30
11	TRAV8–4*05	TRAJ40*01	VVPTTSGTYKYI	TRBV9*01	TRBD2*02	TRBJ2–3*01	ASSTGGGKTDTQY	4	4.65
12	TRAV39*01	TRAJ42*01	AVEDWGGSQGNLI	TRBV19*01	TRBD2*01	TRBJ2–1*01	ASSPASVGQEQF	5	2.33

*TCR* T-cell receptor, *CDR3* Complementarity-determining region, *V* Variable, *D* Diversity, *J* Joining, *TRAV* T-cell receptor Vα, *TRBV* T-cell receptor Vβ

*subtypes of TCR genes

To optimize expression of introduced TCR in T cells, TCR was constructed in a β/α chain order and constant regions was replaced by mouse counterparts modified with interchain disulfide bond and hydrophobic substitution as previously described (Fig. 4c) [17, 25]. The first ranked TCR was lentivirally transduced into PBLs to generate TCR-Ts with transduction efficiency of more than 50% (Fig. 4d).

The ability of these TCR-Ts to specifically identify and mediate effector functions in response to autologous tumor cells in vitro was evaluated with cytokine production and T cell cytotoxicity assays. The TCR-Ts showed high levels of IFN-γ secretion and specific cytotoxicity to ATCs (Fig. 4e, f and Additional file 1: Figure S5). Besides, we found that TCR-Ts displayed significantly diminished levels of IFN-γ with HLA class I blocking antibodies, which indicated the recognition effect of TCR-Ts was mainly restricted by HLA class I presentation (Additional file 1: Figure S4). To further explore the potential cytotoxicity of TCR-Ts to mediate regression of tumor in vivo, a single intravenous injection of TCR-Ts with intraperitoneal DAC was administered into patient-derived xenograft (PDX) models [18, 19]. Of note, administration of TCR-Ts induced a statistically significant regression of tumors than untreated group, untransduced T cells treated group or DAC treated group (Fig. 4g, *p* < 0.001). These findings revealed that tumor-reactive TCR-Ts could exert an antitumor activity against ESCC in vivo and in vitro*.*

## Discussion

Although the adoptive transfer of TILs could mediate regression of metastatic melanoma, most patients with metastatic epithelial cancers did not respond to this therapy [29]. One potential contributing factor could be that TILs used for treatment not only underwent extensive in vitro expansion, but also were usually highly differentiated and exhausted cells with impaired efficacy and limited proliferative potential [30]. Therefore, TCRs isolated from exhausted tumor-specific TILs are introduced into PBLs without impaired immune function to generate tumor-reactive TCR-Ts, which could show stronger anti-tumor response compared with TILs.

In this study, we found phenotype and effect function of TIL fragments derived from different tumor sites were spatially heterogeneous. Of four TIL fragments, only TIL-F1 could specifically identify and kill autologous tumor cells. Therefore, we enriched tumor-reactive T cells from TIL-F1 by in vitro repeated stimulation of autologous tumor cells. Subsequently, we isolated tumor-specific CD8^+^ CD137^+^ T cells from pre- and post-stimulated TIL-F1 co-cultured with tumor cells and identified their most dominant TCRs by single-cell TCR sequencing. Then this TCR was introduced into donor PBLs to generate tumor-reactive TCR-Ts, which specifically identified and killed autologous tumor cells in vivo and in vitro.

The percentages of effector-memory and effect T cells were highest in all four TIL fragments and moreover all four TIL fragments had different expression levels of PD1, which indicated different levels of impairment on proliferation ability and functional activity of all four TIL fragments. Therefore, although TIL-F1 specifically identified autologous tumor cells, their effect function could be impaired [31]. Hence, TCR isolated from tumor-reactive and exhausted TILs was introduced into PBL without impaired immune function to generate tumor-reactive TCR-Ts, which could show stronger anti-tumor efficacy compared with matched TILs.

Recently, most studies demonstrated that TILs were cultured with autologous dendritic cells pulsed with neoantigens to enrich tumor-reactive TILs and isolate their TCRs, but only less than 1% of all somatic mutations could induce T-cell immune response in epithelial cancers [7]. Moreover, this approach was challenged by a recent study reporting that ~ 90% of tumor specific antigens were derived from noncoding regions instead of coding regions [8]. In addition, except for neoantigens, there could be many other tumor-specific antigens, such as phosphoprotein, glycoproteins, and glycolipids [32]. Thus, we speculated that autologous tumor cells could be an important source of tumor antigens and greatly expand tumor-specific T cells. In fact, it was reported that TILs could specifically identify and kill autologous tumor cells [9–11], and moreover our study demonstrated that TIL-F1 cultured with autologous tumor cells was activated and expanded.

CD137 is expressed on T cells recently activated by TCRs engagement, and expression of CD137 on T cells was used to identify and isolate tumor-specific T cells from PBLs and TILs [12, 13]. Therefore, in the study we used CD137 upregulation after ATCs-stimulation to enrich tumor-reactive T cells and isolated their most dominant TCR. And then we found that PBLs modified by the most dominant TCR could identify and kill autologous tumor cells in vivo and in vitro. These findings indicated that autologous tumor cells could be used to enrich tumor-reactive CD137^+^ T cells of which TCRs could be applied for construction of TCR-Ts for patients with ESCC.

Since our study only enrolled one patient, it is difficult to comprehensively evaluate efficacy and success rates of obtaining tumor-reactive TILs and corresponding TCR-T, and thus we planned to enroll more patients to validate efficacy of our approach in the future study. In addition, since our current approach is limited by low-throughput single-cell RT-PCR and Sanger sequencing method, in the future study, we attempted to screen multiple tumor-reactive TCRs from TILs stimulated with ATCs using high-throughput single-cell RNA sequencing method [33, 34].

## Conclusion

We report a strategy based on stimulation with autologous tumor cells and single cell sorting of CD137^+^ T cells for induction of tumor-reactive T cells, isolation of TCRs and construction of TCR-Ts for ESCC, which is especially important for patients without prior knowledge of the specific epitopes and might be applied for other cancers.

## Additional files

Additional file 1: **Figure S1.** Frequency of CD3^+^ T cells in all four TIL fragments. Gated on live CD3 positive population. **Figure S2.** Memory phonotypic characterizations of all four TIL fragments from ESCC patient. **Figure S3.** General view of location and number of cells sorted into 96-well PCR plate. **Figure S4.** HLA-I blocking experiment of TCR-T cells targeting autologous tumor cells by IFNγ-ELISA. **Figure S5.** IFNγ ELISA for TCR-T cells targeting ATCs pretreated with DAC cocktails or not. (DOCX 314 kb)

Additional file 2: **Table S1.** Clinical characteristics of the patient and donors. **Table S2.** Sequences of the primers used for single cell TCRα/β RT-PCR. **Table S3.** Contents of 2-step single-cell RT-PCR. (XLSX 20 kb)

## Data Availability

The datasets used and analysed during the current study are available from the corresponding author on reasonable request.

## Abbreviations

ACT: Adoptive cell therapy
ATCs: autologous tumor cells
CDR3: complementarity determining region
CFSE: carboxyfluorescein succinimidyl amino ester
ELISA: enzyme-linked immunosorbent assay
ELISPOT: enzyme-linked immunospot
ESCC: esophageal squamous cell cancer
HLA: human leukocyte antigen
IFN-γ: interferon γ
PBL: peripheral blood lymphocytes
PD-1: programmed cell death protein-1
PI: propidium iodide
TCR: T cell receptor
TCR-Ts: T cell receptor-engineered T cells
TILs: tumor infiltrating lymphocytes
