# Supplementary material for: Isolation of T cell receptor specifically reactive with autologous tumour cells from tumour-infiltrating lymphocytes and construction of T cell receptor engineered T cells for esophageal squamous cell carcinoma
Source: J Immunother Cancer. 2019 Aug 28;7:232. doi: 10.1186/s40425-019-0709-7 (PMC6714102; doi:10.1186/s40425-019-0709-7)
Supplement: Supplementary file 1 — Figure S1. Frequency of CD3+ T cells in all four TIL fragments. Gated on live CD3 positive population. Figure S2. Memory phonotypic characterizations of all four TIL fragments from ESCC patient. Figure S3. General view of location and number of cells sorted into 96-well PCR plate. Figure S4. HLA-I blocking experiment of TCR-T cells targeting autologous tumor cells by IFNγ-ELISA. Figure S5. IFNγ ELISA for TCR-T cells targeting ATCs pretreated with DAC cocktails or not. (DOCX 314 kb) [file 40425_2019_709_MOESM1_ESM.docx]

| 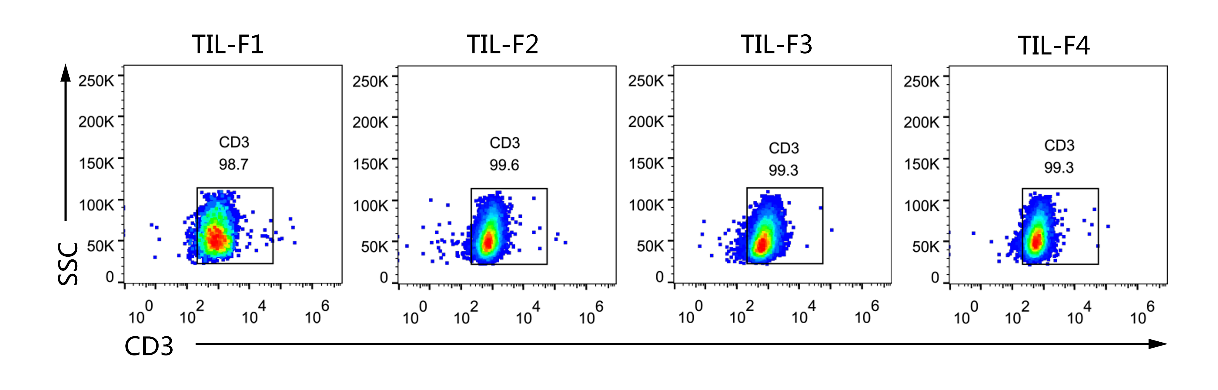 |
| --- |

**Supplementary Figure 1.** Frequency of CD3^+^ T cells in all four TIL fragments. Gated on live CD3 positive population.

| 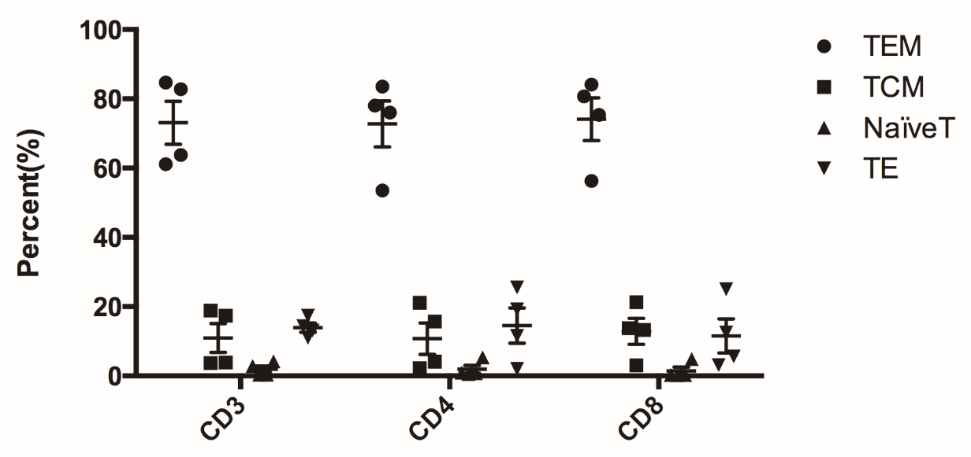 |
| --- |

**Supplementary Figure2.** Memory phonotypic characterizations of all four TIL fragments from ESCC patient. TEM, effector memory T cells; TCM, central memory T cells; Naïve T, Naïve T cells; TE, effector T cells.

| 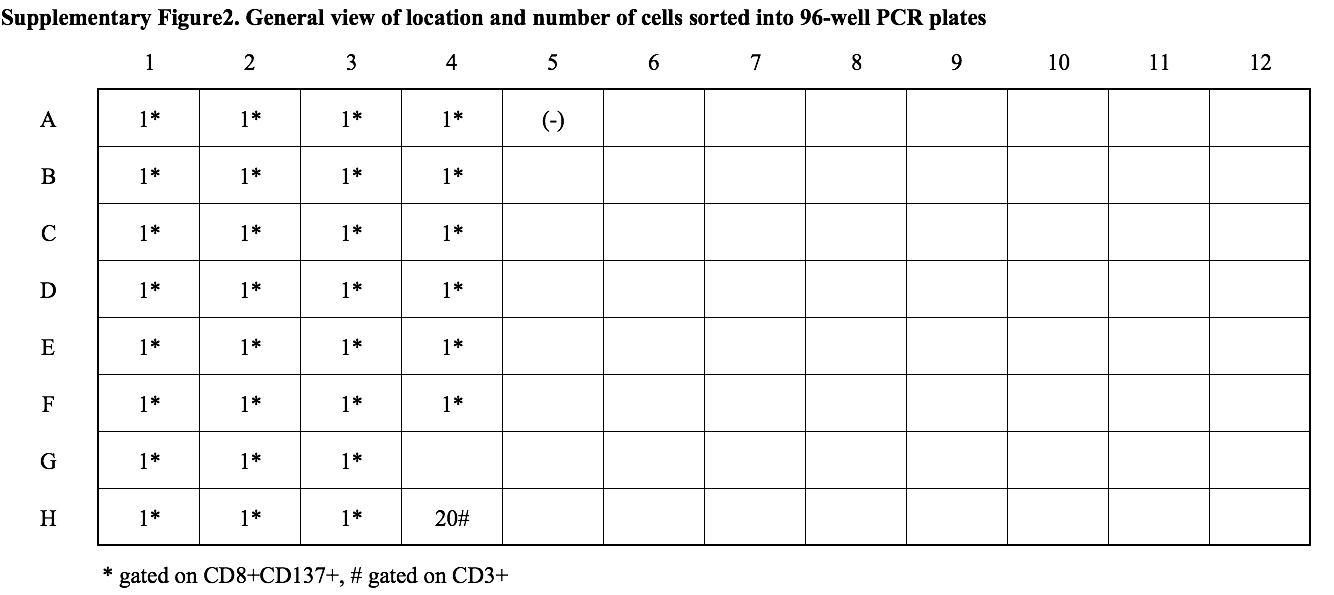 |
| --- |

**Supplementary Figure 3.** General view of location and number of cells sorted into 96-well PCR plate.

|  |
| --- |

**Supplementary Figure 4.** HLA-I blocking experiment of TCR-T cells targeting autologous tumor cells by IFNγ-ELISA. TCR-T cells were co-incubated with or without autologous tumor cells at an E: T ratio of 2:1 in a standard 24-hour IFNγ-ELISA. Parallel co-culture wells were incubated with HLA class I antibody (clone W6/32). TNC, negative control T cells; TCR-T, TCR-engineered T cells; ATC, autologous tumor cell. (***p*<0.01, Student paried *t* test)

| 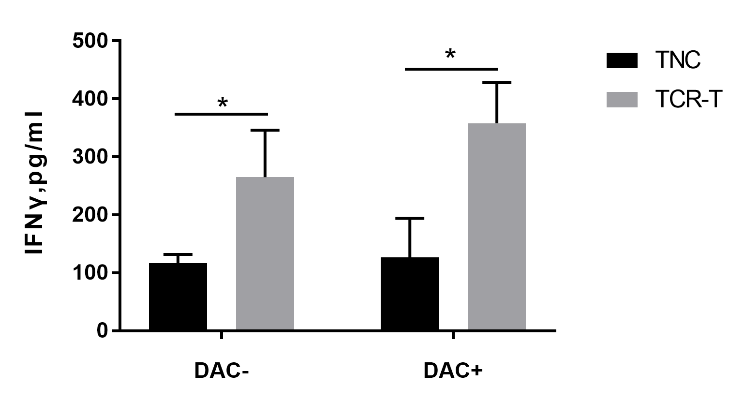 |
| --- |

**Supplementary Figure 5.** IFNγ ELISA for TCR-T cells targeting ATCs pretreated with DAC cocktails (DAC, IFNγ and TNFα) or not. DAC-, ATCs untreated with DAC cocktails; DAC+, ATCs pre-treated with DAC cocktails.
